# Spatiotemporal patterns of particulate matter (PM) and associations between PM and mortality in Shenzhen, China

**DOI:** 10.1186/s12889-016-2725-6

**Published:** 2016-03-02

**Authors:** Fengying Zhang, Xiaojian Liu, Lei Zhou, Yong Yu, Li Wang, Jinmei Lu, Wuyi Wang, Thomas Krafft

**Affiliations:** 1 China National Environmental Monitoring Centre, Beijing, 100012 China; 2 CAPHRI School of Public Health and Primary Care, Maastricht University, Maastricht, The Netherlands; 3 Institute of Geographic Sciences and Natural Resources Research, Chinese Academy of Sciences, Beijing, 100101 P. R. China; 4 Shenzhen Center for Disease Control and Prevention, Shenzhen, 518055 China; 5 Department of Engineering and Safety, University of Tromsø, N-9037 Tromsø, Norway; 6 Institute of Environment Education and Research, Bharati Vidyapeeth University, Pune, 411043 India

**Keywords:** Temporal-spatial patterns, Particulate matter, Mortality, Shenzhen

## Abstract

**Background:**

Most studies on air pollution exposure and its associations with human health in China have focused on the heavily polluted industrial areas and/or mega-cities, and studies on cities with comparatively low air pollutant concentrations are still rare. Only a few studies have attempted to analyse particulate matter (PM) for the vibrant economic centre Shenzhen in the Pearl River Delta. So far no systematic investigation of PM spatiotemporal patterns in Shenzhen has been undertaken and the understanding of pollution exposure in urban agglomerations with comparatively low pollution is still limited.

**Methods:**

We analyze daily and hourly particulate matter concentrations and all-cause mortality during 2013 in Shenzhen, China. Temporal patterns of PM (PM_2.5_ and PM_10_) with aerodynamic diameters of 2.5 (10) μm or less (or less (including particles with a diameter that equals to 2.5 (10) μm) are studied, along with the ratio of PM_2.5_ to PM_10_. Spatial distributions of PM_10_ and PM_2.5_ are addressed and associations of PM_10_ or PM_2.5_ and all-cause mortality are analyzed.

**Results:**

Annual average PM_10_ and PM_2.5_ concentrations were 61.3 and 39.6 μg/m^3^ in 2013. PM_2.5_ failed to meet the Class 2 annual limit of the National Ambient Air Quality Standard. PM_2.5_ was the primary air pollutant, with 8.8 % of days having heavy PM_2.5_ pollution. The daily PM_2.5_/PM_10_ ratios were high. Hourly PM_2.5_ concentrations in the tourist area were lower than downtown throughout the day. PM_10_ and PM_2.5_ concentrations were higher in western parts of Shenzhen than in eastern parts. Excess risks in the number of all-cause mortality with a 10 μg/m^3^ increase of PM were 0.61 % (95 % confidence interval [CI]: 0.50–0.72) for PM_10_, and 0.69 % (95 % CI: 0.55–0.83) for PM_2.5_, respectively. The greatest ERs of PM_10_ and PM_2.5_ were in 2-day cumulative measures for the all-cause mortality, 2-day lag for females and the young (0–65 years), and L02 for males and the elder (>65 years). PM_2.5_ had higher risks on all-cause mortality than PM_10_. Effects of high PM pollution on mortality were stronger in the elder and male.

**Conclusions:**

Our findings provide additional relevant information on air quality monitoring and associations of PM and human health, valuable data for further scientific research in Shenzhen and for the on-going discourse on improving environmental policies.

## Background

Airborne particulate matter (PM) consistently associated with adverse health effects at current levels of exposure in urban populations [1–4]. Air pollution has serious direct and indirect effects on public health in China [2, 5–8]. PM with aerodynamic diameters less than 2.5 μm (PM_2.5_) has become the fourth prominent threat to the health of Chinese people [9].

The range of adverse health effects of air pollution is broad [2, 10, 11]. Susceptibility to pollution may vary depending on overall health condition and age [5, 6, 12–14]. Risk of various effects has been shown to increase with exposure, but there is little evidence to suggest a threshold below which no adverse health effects can be anticipated [15, 16]. The lowest concentration at which such effects begin to manifest is not much greater than the background concentration, which has been estimated at 3–5 μg/m^3^ for PM_2.5_ in the United States and western Europe [15]. Most studies on air pollution exposure and its effects on human health in China have focused on heavily polluted cities or mega-cities [8, 17–19], whereas studies on cities with relatively low air pollutant concentrations are rare.

Shenzhen is a major coastal city with a population of some 15 million. It is situated within the Pearl River Delta (PRD) and Guangdong Province, immediately north of Hong Kong. Shenzhen has become China’s most crowded city and is the fifth most densely populated city in the world, with a population density of 17,150 per square kilometre. Shenzhen is listed as the fourth most important economic centre among Chinese cities. As China’s first and still one of the most successful special economic zones the city has an important position in the PRD region and the country. Compared with other cities in China, air quality in Shenzhen is high. Nevertheless, the city has been experiencing elevated levels of PM pollution in recent years because of rapid economic development [20]. As one of the first-stage cities implementing the National Ambient Air Quality Standard (GB3095-2012) in 2013, Shenzhen provided real-time hourly monitoring concentrations of air pollutants to the general public since January 1 2013. According to air quality monitoring data from the China National Environmental Monitoring Center (CNEMC), respective annual average concentrations of PM_10_ and PM_2.5_ were 61.3 and 39.6 μg/m^3^ in Shenzhen in 2013. The annual average PM_10_ concentration was higher than in 2012 (52 μg/m^3^). However, comprehensive studies on PM in Shenzhen have been rare and there have been no systematic investigations of PM spatiotemporal patterns in Shenzhen.

We carried out a time-series analysis on daily and hourly PM concentrations and daily number of all-cause mortality (excluding accidental deaths) during the first year (2013) of National Ambient Air Quality Standard implementation in Shenzhen. Daily and hourly patterns of PM_2.5_ and PM_10_ were summarized and the daily PM_2.5_/PM_10_ ratio was calculated. Spatial distributions of PM_10_ and PM_2.5_ were investigated. Associations of PM and all-cause mortality were analysed and the susceptibility differentiated according to gender and age were addressed. The objectives were to provide daily/hourly PM_10_ and PM_2.5_ monitoring information for Shenzhen during 2013 to the general public and scientific researchers, investigate spatiotemporal characteristics of PM_10_ and PM_2.5_, evaluate changes of PM_2.5_/PM_10_ ratio, and to discover potential relationships between daily exposure to PM_2.5_ or PM_10_ and all-cause mortality.

Rang of health effects of air pollution was broad [2, 10, 11]. Susceptibility to pollution may vary with health or age [5, 6, 12–14]. Risk of various effects has been shown to increase with exposure, but there is little evidence to suggest a threshold below which no adverse health effects can be anticipated [15, 16]. The lowest concentration at which such effects begin to manifest is not much greater than the background concentration, which has been estimated at 3–5 μg/m^3^ for PM_2.5_ in the United States and western Europe [15]. Most studies on air pollution exposure and its effects on human health in China have focused on heavily polluted cities or mega-cities [8, 17–19], whereas studies on cities with relatively low air pollutant concentrations are rare.

## Methods

### Study area

Shenzhen is in southern China, 113°46–114°37E and 22°27–22°52 N, with an area of 1991.64 km^2^. There is a subtropical oceanic climate, with warm temperatures and abundant rainfall. Annual average temperature is 22.4 °C. The monthly average temperature in January is 15.4 °C, and 28.9 °C in July.

### Data sources

#### Mortality data

All non-accidental mortality data for calendar year 2013 were obtained from death certificates recorded at the Shenzhen Center for Disease Control and Prevention. In the death registry, causes are coded by the International Classification of Disease revision 10 (ICD10).

#### Air pollutant monitoring data

**Fig. 1:**
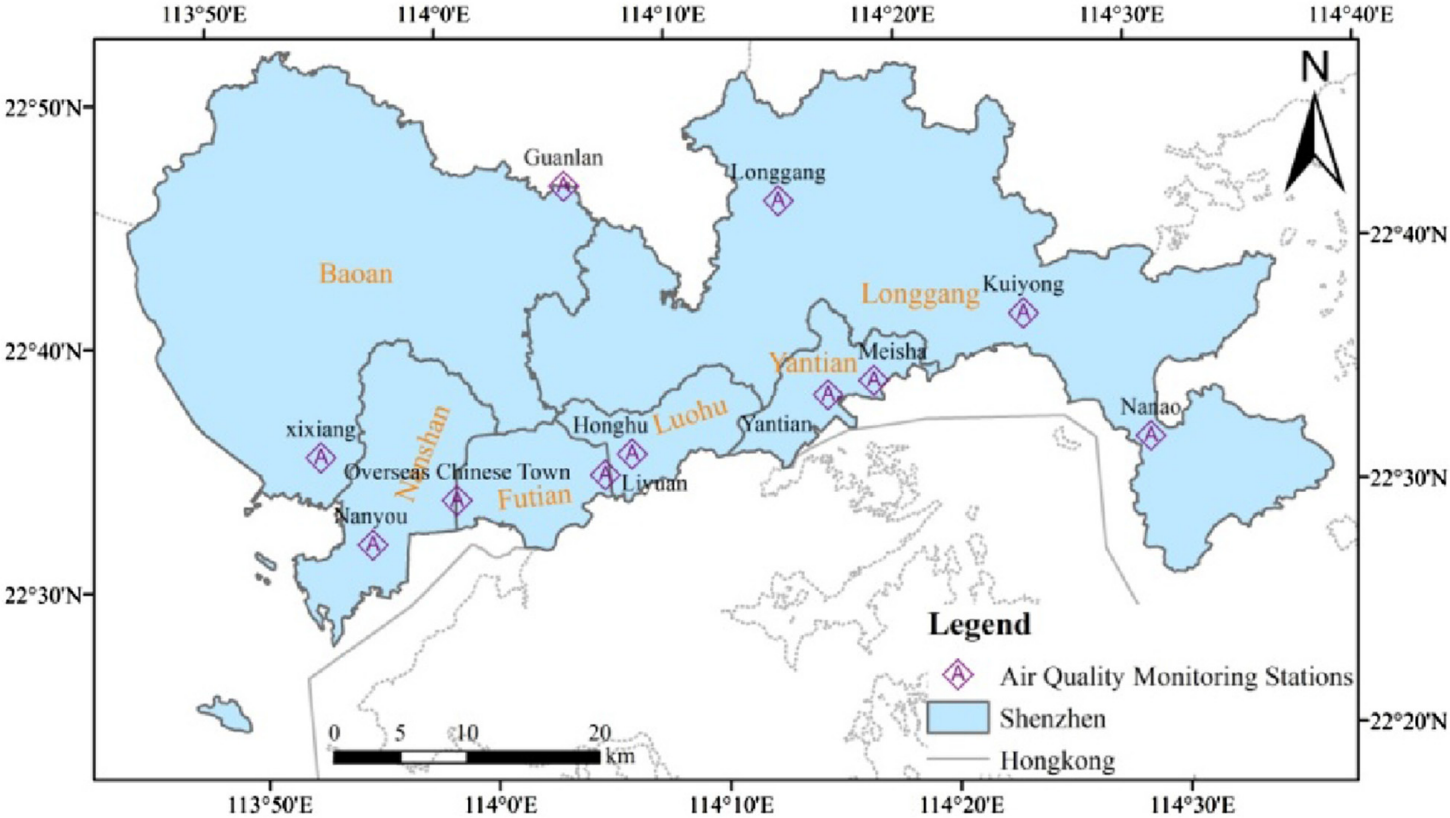
Distribution of 11 state-controlled air quality monitoring sites in Shenzhen

Daily air quality monitoring data were provided by the Shenzhen Environmental Monitoring Center and CNEMC. Daily PM_10_ and PM_2.5_ concentrations were derived from the average of available hourly data measured at 11 state-controlled monitoring stations across Shenzhen, the locations of which are presented in Fig. 1. According to Technical regulation for ambient air quality assessment (on trial) (HJ 663—2013), when calculating daily means of a city, at least 75 % hourly concentrations from the monitoring stations of the city had to be available in a single day. If more than 25 % of the data in a monitoring station was missing in the whole study period, the entire station would be excluded. According to technical guidelines of the Chinese government, these locations must not be in the immediate vicinity of traffic intersections or major industrial polluters, and should be sufficiently distant from any other emission sources. Thus, the monitoring data reflect the general background urban air pollution level in our study area.

To discern spatiotemporal changes of hourly PM_2.5_ and PM_10_ concentrations, we acquired hourly monitoring data from 1 January through 30 November 2013 at two state-controlled monitoring stations. These stations were downtown (Huaqiaocheng, HQC) and in a tourist area (Nan’ao, NA). Hourly PM_2.5_ and PM_10_ monitoring data were from the National Real-Time Air Quality Monitoring Data Publishing Platform developed by CNEMC, which is publicly accessible via the website http://113.108.142.147:20035/emcpublish/.

#### Meteorological data

To control for effects of weather on mortality, meteorological data (temperature, relative humidity, barometric pressure and wind speed) were obtained from the Meteorological Bureau of Shenzhen Municipality. The weather data was monitored at a weather station belonging to that bureau. The monitoring standard is consistent with the international WMO (World Meteorological Organization) standard. There were no missing meteorological data.

### Data analysis

#### Statistical analysis

Spearman correlation coefficients were used to reflect the relationship between PM and meteorological factors during the study period.

#### Spatial analysis

In the Macroscopic regional scale, spatial distribution of PM_2.5_ concentration follow the basic assumption of ‘the first law of geography’, namely the regional concentrations in nearby areas are more similar than in the more distant areas. Therefore, inverse distance weighted model (Inverse Distance Weighted, IDW) interpolation analysis was used to analyze spatial distributions of PM_2.5_ and PM_10_.

#### Associations between daily concentration of PM and mortality

Consistent with other time-series studies [21, 22], we used a generalized additive model (GAM) with penalized splines to analyze mortality, PM, and confounding factors (calendar time, day of week, temperature, barometric pressure, wind speed and humidity). Because the daily mortality number was small and typically followed a Poisson distribution [23, 24], the core analysis was via a GAM with log link and Poisson error that accounted for smooth fluctuations of that number.

In preparation for conducting the model analyses, we conducted two steps in the procedure of the model building and model fit: development of the best base model (without a pollutant) and development of the main model (with a pollutant). The latter is achieved by adding the PM to the final cause-specific best base model, assuming a linear relationship between the logarithmic mortality number and PM concentration.

First, we constructed the basic pattern of mortality number excluding PM. We incorporated smoothed spline functions of time and weather conditions, which can include non-linear and non-monotonic links between mortality and time/weather conditions, offering a flexible modelling tool. Day of the week was also included in the basic models.

After we established the basic models, we introduced the PM and analyzed their associations with mortality. To compare the relative quality of the mortality predictions across these non-nested models, Akaike’s Information Criterion (AIC) was used as a measure of how well the model fitted the data. Smaller AIC values indicate the preferred model. Briefly, we fitted the following log-linear generalized additive models to obtain the estimated pollution log-relative rate β in the study district:

$$ \log \left[\mathrm{E}\left(\mathrm{Y}\mathrm{t}\right)\right]=\upalpha +{\displaystyle \sum_{\mathrm{i}=1}^{\mathrm{q}}}\upbeta \mathrm{i}\left(\mathrm{Xi}\right)+{\displaystyle \sum_{\mathrm{j}=1}^{\mathrm{p}}}\mathrm{f}\mathrm{j}\left(\mathrm{Z}\mathrm{j},\mathrm{d}\mathrm{f}\right)+\mathrm{W}\mathrm{t}\left(\mathrm{week}\right) $$

Here E(Yt) represents the expected number of mortality at day t; β represents the log-relative rate of mortality associated with an unit increase of PM; Xi indicates the concentrations of pollutants at day t; Wt(week) is the dummy variable for day of the week. ${\displaystyle {\sum}_{\mathrm{j}=1}^{\mathrm{p}}}\mathrm{f}\mathrm{j}\left(\mathrm{Z}\mathrm{j},\mathrm{d}\mathrm{f}\right)$ is the non-parametric spline function of calendar time, temperature, barometric pressure, wind speed and humidity. A detailed introduction to GAM is given in Wood [24]. We initialized the df as 7 df/year for time, 3 df for temperature, barometric pressure, wind speed and humidity [25].

Results were expressed as excess risk (ER) in mortality number per 10 μg/m^3^ increases in PM concentrations (ER = (e^βxΔC^-1) × 100, where Δ*C* is the incremental PM amount, which was 10 μg/m^3^ here for comparison with similar studies in other locations of China).

Values of *p* < 0.05 were considered statistically significant.

We also examined PM effects with different lag (L) structures of single-day (distributed lag; L0–L3) and multi-day (moving average lag; L01–L03) lags. Here, a lag of 0 day (L0) corresponds to current-day pollution and a lag of 1 day to the previous-day concentration. In multi-day lag models, L03 corresponds to a 4-day moving average pollutant concentration of the current and previous 3 days [26, 27]. Meteorological factors used in the lag models (distributed and moving average) were from current-day data. While running the models we also considered lags of more than three days for each of the pollutants, but very few associations were identified and these results have been excluded from further analyses.

#### Software used

Temporal changes of PM_2.5_ and PM_10_ were summarized by Origin 9.0 software, and their spatial differences were presented by ArcGIS 10.2 using Inverse distance weighted (IDW). Other statistical analyses were conducted in R3.1.0, and MGCV package in R3.1.0 was used for the GAM analysis.

## Results

### Descriptive results

Table 1 summarizes annual means and percentages of daily mortality number, PM_10_ and PM_2.5_ concentrations, and meteorological factors for Shenzhen in 2013.

**Table 1:** Statistical characteristics of air pollutants, meteorological factors, and daily mortality number

Items	Average	SD	Min	25 %	Mid	75 %	Max
All-cause mortality^a^	32.7	6.5	9	28	32	36	51
Male^a^	20.5	4.9	5	17	20	24	36
Female^a^	12.1	3.7	1	10	12	14	25
Young (0–65years)^a^	17.6	4.5	3	15	17	20	32
Elder (> 65 years)^a^	15.1	4.4	4	12	15	18	29
Temperature (°C)	23.1	5.2	9.8	19.4	24.2	27.7	31.2
Humidity (%)	74.8	15.6	24	67	78	87	100
Pressure (hPa)	1005.2	6.2	986.8	1000.5	1005.1	1010.8	1019.2
Wind speed (m/s)	2.1	0.8	0.3	1.6	2	2.5	5.5
PM_10_ (μg/m^3^)	61.3	33.5	10	34	53	81	179
PM_2.5_ (μg/m^3^)	39.6	24.8	9	20	35	52	135
PM_2.5_/PM_10_ (%)	62.4	10.7	37	54.4	63.2	69.6	100

^a^ daily number. Pressure is barometric

During the study period, mean daily temperature and humidity were 23.1 °C and 74.8 %, respectively. Mean daily temperature was 9.8–31.2 °C and mean daily humidity ranged from 24–100 %, reflecting the subtropical oceanic climate of Shenzhen.

Annual average concentrations were 61.3 μg/m^3^ for PM_10_ and 39.6 μg/m^3^ for PM_2.5_. Averages were higher than median values for the two air pollutants. PM_2.5_/PM_10_ ratios ranged from 37.0 to 88.3 %, with a mean of 62.4 %.

A total mortality of 11,919 people from all causes was observed in 2013. Among these, 7494 were male and 4425 female. There were 6421 people in the 0–65 year age group and 5498 in the 65+ group. The daily number of all-cause mortality was between 9 and 51.

Spearman correlation coefficients for PM and meteorological factors are presented in Table 2. Significant positive correlations were found between PM and barometric pressure. Temperature was negatively and significantly correlated with PM. Similar patterns of correlations were also found for humidity and wind speed.

**Table 2:** Spearman correlation coefficients between air pollutants and meteorological factors

	Temperature	Humidity	pressure	Wind speed	PM_10_	PM_2.5_
Temperature	1	0.250^a^				
Humidity	0.250^a^	1				
Pressure	−0.831^a^	−0.516^a^	1			
Wind speed	0.006	−0.048	−0.054	1		
PM_10_	−0.475^a^	−0.624^a^	0.578^a^	−0.188^a^	1	
PM_2.5_	−0.559^a^	−0.556^a^	0.624^a^	−0.170^a^	0.973^a^	1

^a^Correlation significant at 0.01 level (2-tailed test). Pressure is barometric

### Temporal changes

#### Daily concentrations of PM_10_ and PM_2.5_

**Fig. 2:**
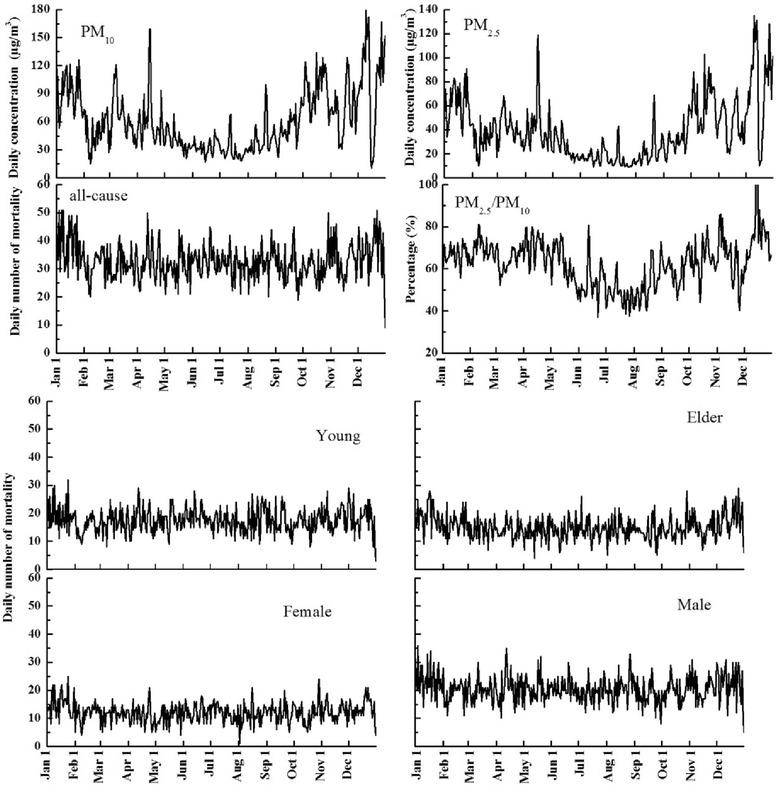
Daily concentrations of PM_10_ and PM_2.5_ and daily number of mortality

Figure 2 shows temporal characteristics of PM_10_ and PM_2.5_. Daily PM_10_ and PM_2.5_ concentrations showed significant similar temporal trends, with relatively high levels during October–December and low levels for May–September.

Daily PM_10_ concentrations were 10–179 μg/m^3^ with an average of 61.3 μg/m^3^, and PM_2.5_ concentrations were 9–135 μg/m^3^ with an average of 39.6 μg/m^3^. PM_10_ and PM_2.5_ concentration ranges were wide, and their maxima were twice the Class 2 limits of National Ambient Air Quality Standard.

#### Ratios of PM_2.5_ to PM_10_

Temporal characteristics of daily PM_2.5_/PM_10_ ratios are shown in Fig. 2. The ratios peaked during December–February and April–May, with low values from June to August, which mean high fine particulate ratio in December–February and April–May. The ratios ranged from 37.0 to 88.3 %, with an average of 62.4 %.

#### Temporal trends of daily mortality number

Daily trends on number of mortality for all-cause, male, female, young and elder are also summarized in Fig. 2. Daily mortality number for all-cause was 9–51 with an average of 33, 5–36 for male with an average of 21, 1–25 for female with an average of 12, 3–32 for young with an average of 18, 4–29 for elder with an average of 15.

### Spatial differences

**Fig. 3:**
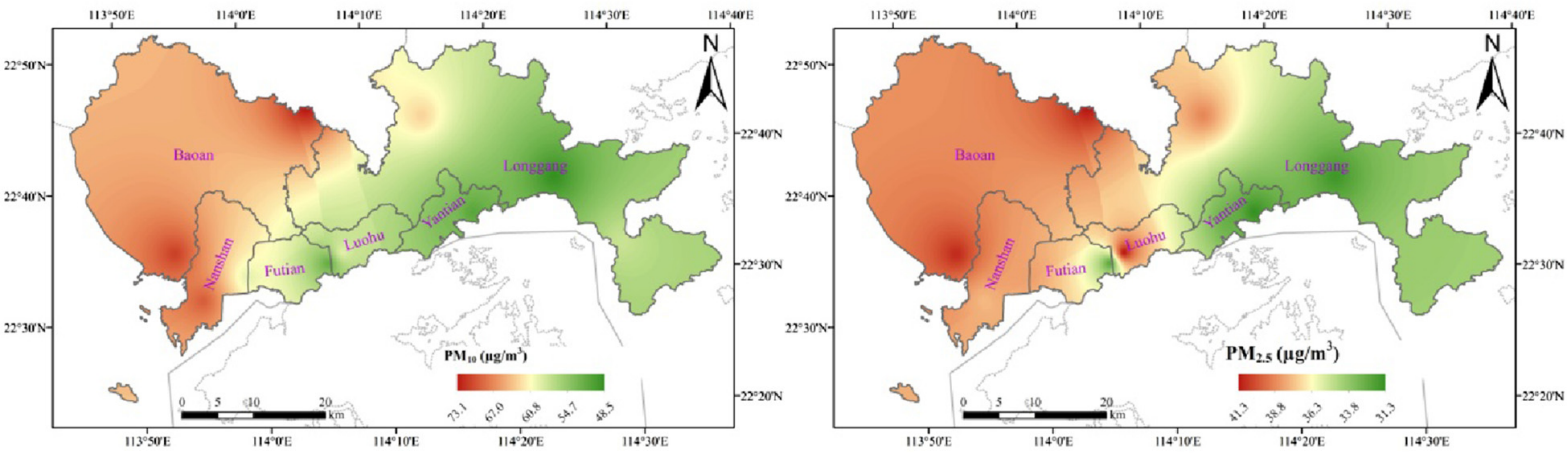
Spatial distributions of PM_10_ and PM_2.5_ in Shenzhen

Figure 3 presents spatial distributions of PM_10_ and PM_2.5_ in Shenzhen during 2013. PM_10_ and PM_2.5_ concentrations were higher in western parts of Shenzhen than in eastern parts. According to National Ambient Air Quality Standard, annual average PM_2.5_ concentrations at five monitoring stations named Nan’ao, Liyuan, Kuiyong, Meisha and Yantian were within Class 2 limits (annual average PM_2.5_ concentration < 35 μg/m3), but exceeded these limits at the other six stations.

To represent spatial differences of PM in Shenzhen more directly, we analysed monthly and hourly concentrations of PM_10_ and PM_2.5_ at HQC and NA sites, in the downtown and tourist areas of Shenzhen, respectively. These two parts of the city serve distinct and quite different urban functions, and differences in air quality might indicate that they are affected by different pollutant emission sources.

#### Monthly differences

**Fig. 4:**
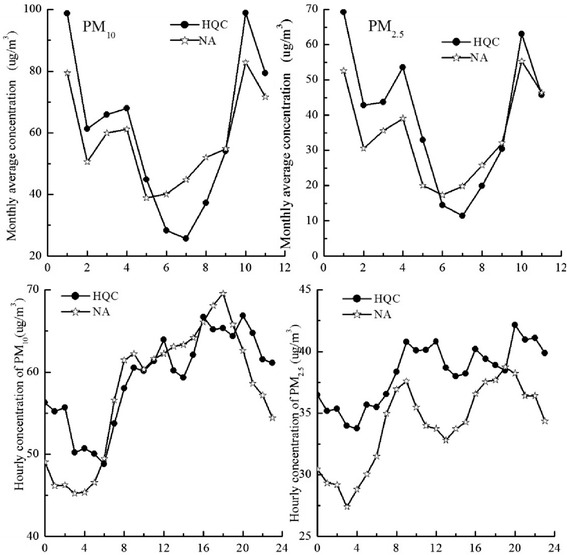
Monthly/hourly PM_10_ and PM_2.5_ concentrations at HQC and NA

Figure 4 shows average monthly PM_10_ and PM_2.5_ concentrations at HQC and NA from January through November. The results show that PM_10_ and PM_2.5_ had similar hourly trends at HQC, which had higher concentrations in January and October and lower concentrations in July. At the NA monitoring station, both PM_10_ and PM_2.5_ had higher concentrations in January and October, and lower concentrations in May for PM_10_ and June for PM_2.5_. Concentrations of PM_10_ at HQC were higher than at NA during January–May and September–November, and lower than at NA during May–September. Concentrations of PM_2.5_ at HQC were higher than at NA during January–June and September–November, and lower than at NA during June–September.

#### Hourly differences

Hourly average PM_10_ and PM_2.5_ concentrations at HQC and NA in 2013 are also presented in Fig. 4. Changes in hourly concentrations of PM_10_ and PM_2.5_ had similar patterns at NA, which had maxima at 20:00 and secondary maxima at 9:00. Minima were from 3:00 to 4:00. Changes in hourly concentration of PM_10_ and PM_2.5_ did not show patterns common with HQC, which may be related to changes of pollution source over a day in the downtown area. Hourly concentrations of PM_2.5_ at NA were lower than at HQC throughout the day, but the hourly PM_10_ concentration did not show this pattern.

### Associations with all-cause mortality

Table 3 presents ER percentages(ERs) (95 % confidence interval [CI]) of daily all-cause mortality number with every 10-μg/m^3^ increase in PM_10_ or PM_2.5_ concentration.

**Table 3:** Excess risk (ER) percentage for daily all-cause mortality number with every 10-μg/m3 increase in PM concentration

Items		All-cause	Female	Male	Elder	Young
		ER (95 % CI)	ER (95 % CI)	ER (95 % CI)	ER (95 % CI)	ER (95 % CI)
PM_10_	L0	0.22(0.12 ~ 0.32)	−0.32(−0.48 ~ 0.17)	0.54(0.42 ~ 0.67)	1.35(1.21 ~ 1.49)	−0.79(−0.92 ~ 0.65)
	L1	0.37(0.28 ~ 0.46)	0.33(0.19 ~ 0.47)	0.40(0.29 ~ 0.51)	1.05(0.92 ~ 1.17)	−0.24(−0.36 ~ 0.12)
	L2	0.51(0.43 ~ 0.60)	0.33(0.21 ~ 0.46)	0.62(0.52 ~ 0.73)	0.85(0.74 ~ 0.97)	0.21(0.10 ~ 0.33)
	L3	−0.02(-0.10 ~ 0.06)	−0.47(−0.59 ~ 0.34)	0.25(0.15 ~ 0.35)	0.41(0.29 ~ 0.53)	−0.41(−0.52 ~ 0.29)
	L01	0.42(0.31 ~ 0.52)	0.05(−0.12 ~ 0.22)	0.64(0.51 ~ 0.77)	1.54(1.39 ~ 1.69)	−0.57(−0.71 ~ 0.42)
	L02	0.61(0.50 ~ 0.72)	0.23(0.06 ~ 0.40)	0.85(0.71 ~ 0.98)	1.55(1.4 ~ 1.71)	−0.22(−0.37 ~ 0.07)
	L03	0.45(0.34 ~ 0.57)	−0.07(−0.25 ~ 0.10)	0.77(0.64 ~ 0.91)	1.39(1.24 ~ 1.55)	−0.38(−0.54 ~ 0.23)
PM_2.5_	L0	0.12(−0.01 ~ 0.24)	−0.35(−0.54 ~ 0.15)	0.40(0.24 ~ 0.56)	1.67(1.48 ~ 1.85)	−1.26(−1.44 ~ 1.09)
	L1	0.46(0.35 ~ 0.58)	0.76(0.58 ~ 0.94)	0.29(0.15 ~ 0.44)	1.39(1.22 ~ 1.55)	−0.37(−0.53 ~ 0.21)
	L2	0.68(0.57 ~ 0.79)	0.75(0.57 ~ 0.92)	0.65(0.51 ~ 0.78)	1.13(0.97 ~ 1.28)	0.28(0.12 ~ 0.43)
	L3	−0.23(−0.34 ~ 0.12)	−0.63(−0.80 ~ 0.45)	0.01(−0.13 ~ 0.15)	0.55(0.39 ~ 0.71)	−0.94(−1.1 ~ 0.79)
	L01	0.41(0.28 ~ 0.55)	0.31(0.10 ~ 0.52)	0.48(0.31 ~ 0.65)	1.92(1.73 ~ 2.11)	−0.92(−1.11 ~ 0.73)
	L02	0.69(0.55 ~ 0.83)	0.62(0.40 ~ 0.84)	0.74(0.56 ~ 0.91)	1.97(1.77 ~ 2.16)	−0.45(−0.64 ~ 0.25)
	L03	0.44(0.29 ~ 0.58)	0.19 (−0.04 ~ 0.42)	0.59(0.41 ~ 0.76)	1.82(1.62 ~ 2.03)	−0.80(−1.00 ~ 0.60)

To identify possible time delay of PM_10_ or PM_2.5_ pollution exposure and daily mortality number, we analyzed lag effects of air pollutants. ER in the all-cause mortality number with a 10 μg/m^3^ increase of pollutants for single-day measures, 1–3 days prior to mortality (L0–L3), and moving averages from day 0 and day 1 to day 3 prior to the mortality are also listed in Table 3. When running the models, lag effects of more than 3 days for PM_10_ and PM_2.5_ were also considered. However, as little to no relationship was found, the results of that analysis were not included. Gender and age differences were also considered. Unlike cities in northern China, temperature differences in Shenzhen were not significant. Therefore, we did not run seasonal models.

The results showed that the greatest ERs of PM_10_ and PM_2.5_ were in 2-day cumulative measures (L02) for the all-cause mortality group, 2-day lag (L2) for females and the young (0–65 years), and L02 for males and the elder (> 65 years). The greatest ERs in the mortality number with a 10-μg/m^3^ increase of PM_10_ were 0.61, 0.33, 0.85, 1.55 and 0.21 % for the all-cause mortality group, females, males, elder and young, respectively. The greatest ERs in the mortality number with a 10-μg/m^3^ increase of PM_2.5_ were 0.69, 0.76, 0.74, 1.97 and 0.28 % for the same respective groups. ERs of males with increases in PM_10_ or PM_2.5_ concentration were greater than those of females, and ERs of the elder were greater than the young with concentration increases of PM_10_ or PM_2.5_.

## Discussion

This study focused on spatiotemporal patterns and possible associations of PM_10_ and PM_2.5_ with all-cause mortality during the first year (2013) of National Ambient Air Quality Standard implementation in Shenzhen, a relatively clean city compared to other cities in China. The objectives were to provide 2013 PM monitoring information of Shenzhen to the general public, to discover possible associations between PM and mortality in a comparatively clean city, and to provide scientific results to researchers in other areas. We also intend to encourage health services and public health policymakers in Shenzhen to consider ideas for real-time public health alerts for air quality, so that vulnerable groups and others affected by air pollution can be appropriately advised. The present study was unique in the following aspects: 1) we analysed daily patterns of PM and air quality during the first year (2013) of National Ambient Air Quality Standard implementation in Shenzhen; 2) based on reliable data sources, hourly/monthly patterns of PM in two functional areas of Shenzhen were addressed; 3) spatial patterns of PM were determined; and 4) to our knowledge, the study is the first to investigate associations between PM and all-cause mortality in Shenzhen.

During 2013, annual average PM_10_ and PM_2.5_ concentrations were 61.3 and 39.6 μg/m^3^, respectively; averages were higher than the median values for the two air pollutants. According to National Ambient Air Quality Standard and the Technical Regulation on Ambient Air Quality Index (on trial) (HJ633-2012), PM_2.5_ was the major air pollutant in Shenzhen, with 104 days as the primary pollutant and 32 days as a “non-attainment” pollutant. The latter indicates 32 days with heavy PM_2.5_ pollution. Annual average PM_10_ and PM_2.5_ concentrations were 108 and 89 μg/m^3^ in Beijing, and 72 and 53 μg/m^3^ in Guangzhou [28]. Compared with heavy PM-polluted cities in China (Beijing, Guangzhou, and others), Shenzhen has good air quality [9, 19, 29]. Shenzhen was ranked 7th among 74 first-stage cities, but its annual average PM_2.5_ concentration exceeded the Class 2 limit of National Ambient Air Quality Standard [28, 30].

The annual average ratio of PM_2.5_ to PM_10_ was 62.4 %, which indicates a high percentage of PM_2.5_ in ambient air pollution of Shenzhen. PM_2.5_/PM_10_ maximized in December–February and April–May, with lower values in June–August. Compared with Beijing the PM_2.5_/PM_10_ ratio in Shenzhen was higher than Beijing autumn normal days and lower than haze days and winter normal days in Beijing; the average PM_2.5_/PM_10_ ratios in Beijing were correspondingly 0.63, 0.32, 0.70, and 0.66 in autumn haze, autumn normal, winter haze and winter normal days, respectively [31]. The PM_2.5_/PM_10_ ratio is 0.575 in Taiwan [32]. These findings may be related to meteorological conditions and pollution sources in the city. PM_2.5_ concentrations can be affected by both local emissions and contributions of meso-scale origin [33]. Further studies on concentrations and ratios of PM at intercity level should be conducted.

Hourly concentrations of PM_10_ and PM_2.5_ had similar patterns in the tourist area (NA monitoring station) but did not have any patterns in common in the downtown area. This may be related to changes of pollution source like traffic emission in downtown over a single day. Hourly concentrations of PM_2.5_ in the tourist area were lower than downtown throughout the day, which may be attributed to more intensive human activities downtown. PM_10_ and PM_2.5_ concentrations were higher in western parts of Shenzhen than eastern parts, which may be related to land use, pollution sources, industrial structure, traffic conditions, and other factors [34]. There should be further study of relationships between driving factors (e.g., spatial distribution of pollutant emissions, pollutant emission intensity, and regional industrial structure) and pollutant concentrations.

Time-series studies estimate that a 10 μg/m^3^ increase in mean 24-hour PM_2.5_ concentration increases the ERs of daily cardiovascular mortality by ~0.4 to 1.0 % [35]. Consistent with other studies [2, 11, 12, 36, 37], we found a statistically significant association between PM_10_ or PM_2.5_ and daily mortality number. There were lag effects in all the study groups, and ERs of PM_2.5_ were greater than PM_10_ in all study groups with concentration increases. ERs in the all-cause mortality number with a 10-μg/m^3^ increase of PM_10_ and PM_2.5_ were 0.61 % (95 % CI: 0.50 %–0.72 %) and 0.69 % (95 % CI: 0.55 %–0.83 %), respectively. A study in Beijing for 2005–2009 showed that a 10 μg/m^3^ increase in PM_2.5_ was associated with a 0.65 % rise in all-cause mortality, whereas the same increase in PM_10_ was associated with an increase of 0.15 % [38]. During 2006–2009 in Guangzhou, increments of 10 μg/m^3^ in PM_10_ were associated with a ER of 1.26 % for total non-accidental deaths, and 1.79 % for cardiovascular deaths [39]. During 2007 to 2009 in Tianjin, the effect estimates per 10 μg/m^3^ increase in PM_10_ concentrations at the moving average of lags 0 and 1 day in high temperature level were 0.62 % for non-accidental mortality [40]. ERs in our study were greater than those reported for Beijing and smaller than those for Guangzhou. Such inter-city variability in ER estimates may have been influenced by a number of factors, such as demographic and socioeconomic variables, culture, air pollution sources, and geographical and weather conditions [39]. Both temperature and particulate air pollution are associated with increased death risk; and extreme high temperature increased the associations of PM_10_ with daily mortality [41]. ERs in the all-cause mortality number with 10-μg/m^3^ increase of PM_10_ and PM_2.5_ were 0.33 and 0.76 % for females, respectively, 0.85 and 0.77 % for males, 1.55 and 1.97 % for the elder, and 0.21 and 0.28 % for the young. Males were more sensitive to PM_10_ or PM_2.5_ concentration changes than females. The elder appeared to be more affected than the young by PM_10_ or PM_2.5_ concentration increase. These findings indicate that PM effects on mortality were stronger among the elderly and on male. Because the seasonal difference was not significant in Shenzhen, we did not consider seasonal associations among PM and mortality in this study. The present study has certain limitations. We considered the target population to be relatively homogeneous and did not consider residence or work location of deaths, owing to a lack of data. Pollutant exposure levels were derived from 11 fixed-site monitoring stations. However, because air pollution varies spatially within a city, averages drawn from these stations may not reflect actual exposure levels. Accurate exposure assessment and a homogeneous target population are important factors to consider in future studies estimating mortality risk from air pollution [39]. Further in-depth studies should require air pollutant composition, pollution emission sources, pollutant emission patterns, time-series of human activity, individual exposure to pollutants, social economy, and human health at the city level.

## Conclusion

During 2013, annual average PM_10_ and PM_2.5_ concentrations were 61.3 and 39.6 μg/m^3^ in Shenzhen. PM_2.5_ failed to meet the Class 2 annual limit of National Ambient Air Quality Standard and was the major air pollutant, with 8.8 % of days having heavy PM_2.5_ pollution. The annual average PM_2.5_/PM_10_ ratio was 62.4 %. Hourly PM_2.5_ concentrations in the tourist area were lower than downtown throughout the day. PM_10_ and PM_2.5_ concentrations were higher in western parts than eastern parts. ERs in the all-cause mortality number increased with PM_10_ and PM_2.5_. PM_2.5_ had higher risks than PM_10_. PM effects on mortality were stronger among male and the elderly. Our findings provide additional information on air quality monitoring and associations between PM and all-cause mortality, and valuable data for scientific research in Shenzhen. It also contributes to the discussion on further developing environmental health policies in urban China.
